# Organic fertilizer increases pumpkin production by improving soil fertility

**DOI:** 10.3389/fpls.2024.1467931

**Published:** 2024-11-14

**Authors:** Fangfang Ren, Jinxia Zhang, Lin Ding, Rui Zhang, Fuqiang Li, Xuan Li, Tao Zhong, Meng Yin, Runheng Yang, Pengliang Tian, Liangliang Du, Kaiyuan Gan, Tian Yong, Qirun Li, Xingrong Liu

**Affiliations:** ^1^ College of Water Conservancy and Hydropower Engineering, Gansu Agricultural University, Lanzhou, China; ^2^ Rural Water Conservancy Institute, Gansu Academy for Water Conservancy, Lanzhou, China; ^3^ Geological Hazards Prevention Institute, Gansu Academy of Sciences, Lanzhou, China

**Keywords:** organic fertilizer, soil bulk density, soil organic carbon, soil NPK, pumpkin yield

## Abstract

Compared with long-term and continuous application of large amounts of chemical fertilizers, fertilizers with microbial organic nutrient sources can improve soil environment, increase soil fertility and increase crop yield. In view of the current low soil fertility and poor soil environment leading to low crop yield and instability in the arid regions of northwest China, the effects of organic fertilizer with microbial nutrient sources on soil nutrients and pumpkin yield were studied in 2022 and 2023 in this region. The fertilizer application level was used as control factor, with four treatments of low level (L), medium level (M), high level (H), and a conventional fertilizer control (CK). The results showed that the high application level of organic fertilizer was more beneficial to the growth of pumpkin, and the stem diameter, vine length, and leaf area of pumpkin under H treatment were the highest from 2022 to 2023. Compared to CK, the average soil bulk density was significantly decreased by 8.27–18.51% (P< 0.05); the soil organic carbon, available phosphorus, available potassium, and nitrate nitrogen under H treatment were increased by an average of 32.37%, 21.85%, 18.70%, and 36.97%, respectively. Under different organic fertilizer treatments, the pumpkin yield under M treatment was the highest, reaching 30926.18 kg·ha^-1^, followed by H treatment. compared to CK, M and H treatments increased the yield by 25.26% and 7.01%, respectively, and improved water use efficiency by 14.18% and 2.21%, respectively. Redundancy analysis (RDA) of soil nutrients, pumpkin growth dynamics and yield in 2022 and 2023 showed that soil organic carbon, available phosphorus, available potassium, nitrate nitrogen, and water use efficiency were significantly positively correlated with pumpkin yield (P<0.01). In conclusion, H and M treatments can improve soil fertility promote pumpkin growth and development, and ultimately increase pumpkin yield. In summary, medium organic fertilizer level (M=5700 kg·ha^-1^) is recommended as the fertilization scheme for local pumpkin cultivation.

## 1 Introduction

In the current, water scarcity, scarce rainfall, and low soil fertility have been the major limiting factors in achieving high and stable crop yields in the arid areas of Northwest China (Xue et al., 2017; Zhang et al., 2023; Zong et al., 2023). How to improve soil fertility and increase crop yields under limited water resources is a long-term technical challenge in agricultural production of the region (Zhang et al., 2018b; Li et al., 2023). Among them, fertilization played an important role in soil fertility and crop yields, being one of the most fundamental measures to enhance farmland productivity (Li et al., 2020a). However, long-term and sustained application on large amounts of chemical fertilizers could have adverse effects on soil properties, leading to a decrease in fertilizer efficiency (Cui et al., 2020; Santiago et al., 2019), with soil nitrate accumulation and other ecological problems. As an alternative to chemical fertilizers in the process of green and sustainable agriculture development, microbial organic fertilizers could improve soil environment, increase soil fertility, and enhance crop yields (Wei et al., 2016). Hence, there is a current emphasis on researching the impact of organic fertilizers on soil fertility and crop yields is currently a hot topic and focus of farmland studies in the arid areas of Northwest China, which holds significant academic value.

Numerous studies have shown that the increased application of organic fertilizers could improve soil fertility, enhance ecological environment, and increase crop nutrient absorption and utilization efficiency (Wang et al., 2019; Mihoub et al., 2023). Improving plant growth and promoting crop yield played an important role in the sustainable utilization of soil, on the premise that it would not have any adverse effects on the agricultural ecosystem (Iqbal et al., 2019; Fawzy et al., 2016). Organic fertilizers could enhance soil fertility by activating microorganisms, improving soil structure, and increasing soil water retention. Over time, organic fertilizers slowly and continuously release nutrients for crops (Ünlükara et al., 2022). Applying organic fertilizers had natural advantages in improving soil compaction, enhancing soil fertility, promoting crop growth, and increasing crop yield (Zhang et al., 2018a). Substituting some chemical fertilizers with organic fertilizers could significantly increase crop yield (Liu et al., 2021a; Li et al., 2021c; Li et al., 2018). Zhou et al. found that crop yield increased by 26.4–44.6% under organic fertilizer treatment (Zhou et al., 2022). It was also found that organic fertilizers had a promoting effect on crop growth and yield increase (Alharbi et al., 2021; Salman et al., 2023). Soil organic carbon content increased with the application of organic fertilizers (Zhao et al., 2024). Applying organic fertilizers increased soil organic carbon content by 13.30–40.56% (Zhang et al., 2022), and decreased soil bulk density by 4.0–5.6% (Duan et al., 2023). Compared to traditional fertilization methods, organic fertilizers could effectively promote crop growth, significantly increase plant height, stem diameter, and fruit setting rate, and enhance crop yield by 22.0% (Jiang et al., 2022). The application of organic fertilizers could increase the yields of leafy vegetables and fruit vegetables by 76.44% and 41.75%, respectively. Long-term application of organic fertilizers was more beneficial for vegetable yield, and organic fertilizer application in general significantly increased the yield by 44.11% in China (Xiang et al., 2022). The research on the utilization of organic fertilizers is anticipated to significantly improve soil environment, enhance soil fertility in farmland, and carry important practical significance for achieving high and stable yields. This study addresses the challenges of declining soil fertility, decreased crop yields, and low efficiency in fertilizer use resulting from prolonged application of single chemical fertilizer in the regions.

Pumpkin is a crop for both food and vegetable, with rich nutrition and high medicinal value. It also has characteristics of barren resistance, drought resistance, and strong adaptability, one of the suitable crops to increase economic output in the arid regions of Northwest China (Ta et al., 2016; Zang et al., 2017). Soil nutrients play an important role in growth, development, and high yield of pumpkins (Zhang et al., 2020). Bacillus subtilis in organic fertilizers is widely present in soils under various natural conditions. It can enhance the decomposition rate of organic fertilizers, promote the absorption of nutrients to plant roots system, accelerate plant growth, and ultimately increase yield (Wei et al., 2011). In conclusion, most researches on the substitution of organic fertilizers for chemical fertilizers mainly focused on grain crops, while limited studies on its impact on the spatial and temporal distribution and transport of soil nutrients in pumpkin fields, as well as the growth status and yield of pumpkins. Therefore, the present study aims to investigate the influence of organic fertilizer application on pumpkin growth characteristics. It is hypothesized that the impact of organic fertilizer application on the temporal and spatial transport of soil nutrients in pumpkin farmland would be obtained. Additionally, the main soil nutrient factors regulating pumpkin yield and growth characteristics would be identified, with the goal of providing scientific guidance fertilization application to enhance pumpkin productivity in the arid regions of Northwest China.

## 2 Materials and methods

### 2.1 Study site

**Figure 1:**
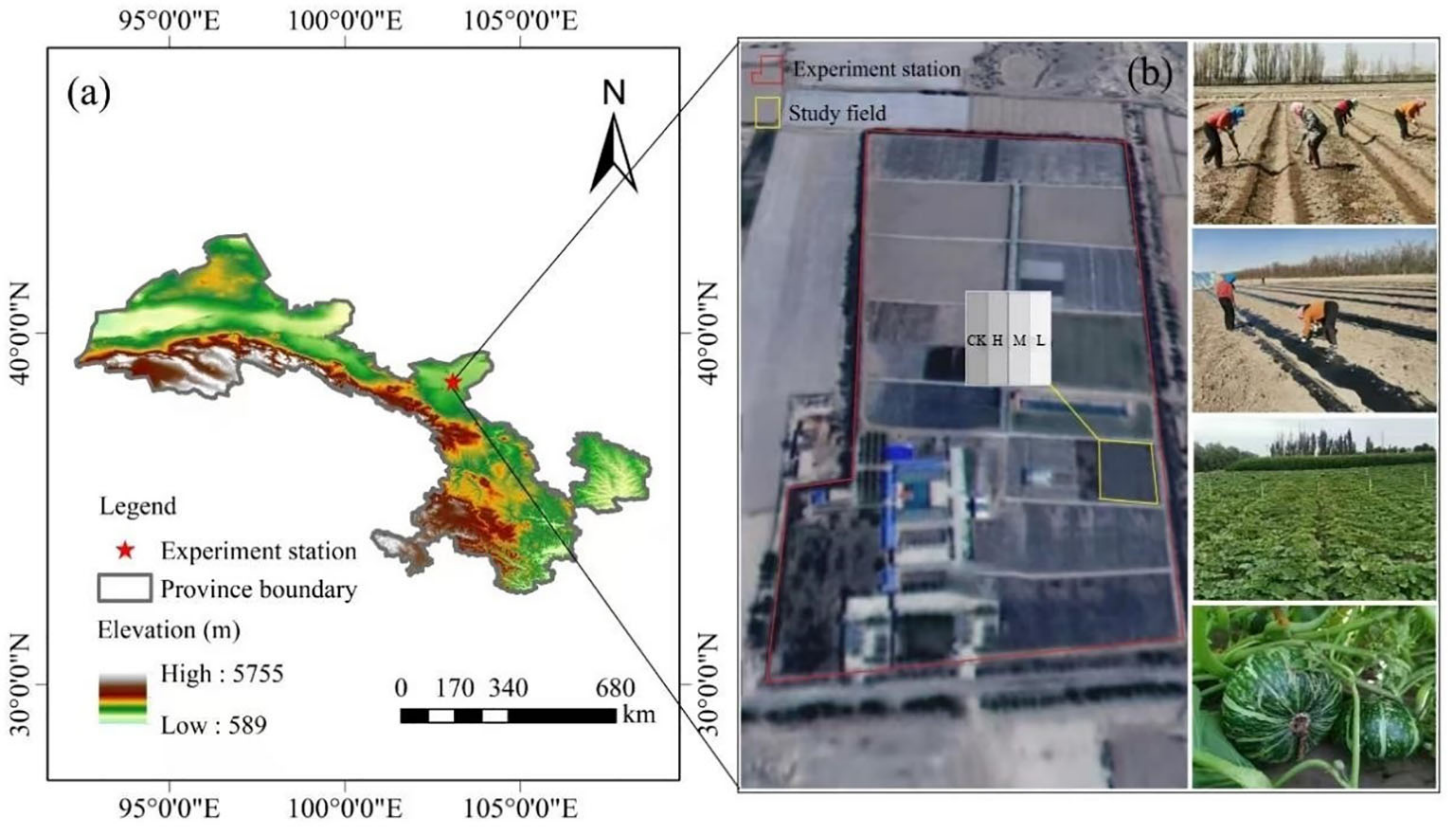
**(A)** Geographical location of the experimental site; **(B)** experimental design and scenes of trial plots establishment, treatment arrangement and field management.

The experiment was conducted at the Minqin Irrigation Experimental Station of Gansu Water Conservancy Science Research Academy in 2022 and 2023. The station is located approximately 13.5 km north of Minqin County, Gansu Province, China (103°05’ E, 38°37’ N), as shown in **Figure 1**. The region is situated at the junction of an oasis and the Tengger Desert, characterized by a typical continental desert climate, with dry conditions, scarce precipitation, high evaporation, abundant wind and sand, and frequent natural disasters. In this region, the annual average air temperature is 7.8°C, with its extreme maximum of 39.5°C and extreme minimum of -27.3°C. Years of average humidity is 45%, annual average precipitation is 110mm, with average evaporation of 2644mm. There are also abundant light and heat resources, with annual sunshine hours of 3028 h, accumulated temperature ≥ 0°C of 3550°C, accumulated temperature ≥ 10°C of 3145°C, the frost-free period of 150 days. And the maximum frozen soil depth is 115cm. The cultivated soil in the experimental area (0–60cm) is clay loam, gradually transitioning to sandy loam below 60 cm, with an average soil bulk density of 1.54 g·cm^-3^. The detailed physical and chemical properties of the experimental field soil were referred in **Table 1**.

**Table 1:** Soil physical and chemical properties in experimental area.

Measuring item	Soil depth (cm)				
	0–20	20–40	40–60	60–80	80–100
bulk density (g·cm^-3^)	1.38	1.48	1.56	1.61	1.68
Field water retention (%)	21.73	22.15	22.87	23.69	24.56
Organic matter (%)	0.8	0.68	0.57	0.34	0.38
Available phosphorus (mg·kg^-1^)	72.06	7.45	2.98	4.58	3.44
Available potassium (mg·kg^-1^)	180	170	190	120	110
Alkaline hydrolyzed nitrogen (mg·kg^-1^)	33	23	18.60	16.10	11.60
Total nitrogen (%)	0.063	0.061	0.054	0.023	0.045

### 2.2 Experimental materials

The test crop is pumpkin, and the test variety is “*Sweet Pumpkin*” (produced by Gansu Delongyuan Agricultural Science and Technology Co., Ltd., CHN). The organic fertilizer for the test is produced by Lanzhou Xindali Water Fertilizer Integration Service Co., Ltd., with effective fertilizer components: N+P_2_O_5_+K_2_O ≥ 18%, effective bacteria (Bacillus subtilis + Bacillus licheniformis) ≥ 0.5 billion/ml, amino acids ≥ 3%.

### 2.3 Experimental design and soil sampling

In the experiment, the organic fertilizer application amount was selected as the control factor, with 4 levels being set at low (L=4500 kg·ha^-1^), medium (M=5700 kg·ha^-1^), high (H=6900 kg·ha^-1^), and control (CK, was the conventional chemical fertilizer application level of local farmers), respectively, replicated 3 times with 12 plots. The fertilizer application was divided into basal fertilizer before sowing and top dressing fertilizer twice during the growth period of pumpkin. At the low, medium, and high levels of organic fertilizer, basal fertilizer was applied of 3900 kg·ha^-1^, 4800 kg·ha^-1^, and 5700 kg·ha^-1^, with top dressing fertilizer at 300 kg·ha^-1^, 450 kg·ha^-1^, 600 kg·ha^-1^ each time, respectively. However, at the CK, base fertilizer of diammonium phosphate 300 kg·ha^-1^ and urea 450 kg·ha^-1^ were applied, with topdressing twice and urea 300 kg·ha^-1^ each time. The experiment was designed according to randomized blocks, with an area of 75 m^2^ (30 × 2.5 m) and furrow irrigation in each plot. Sowing was carried out at a density of 200 cm in furrows, 50 cm in rows, 30 cm in plant spacing, with one furrow and one film in two rows. During the experimental period, the irrigation regime was the same for each treatment throughout the pumpkin growing period. The experimental design protocol is detailed in **Table 2**, the division of the pumpkin growing period, was referred to **Table 3**.

**Table 2:** Experimental treatment design.

Treatment	Base fertilizer dosage (kg·ha^-1^)		Additional fertilizer amount (kg·ha^-1^)		Top dressing times	Irrigation times	Irrigation quota (m^3^·ha^-1^)
L	Solid organic fertilizer	3900	Liquid organic fertilizer	300	2	3	525
M	Solid organic fertilizer	4800	Liquid organic fertilizer	450	2	3	525
H	Solid organic fertilizer	5700	Liquid organic fertilizer	600	2	3	525
CK	Diammonium phosphate Urea	300 450	Urea	300	2	3	525

**Table 3:** Division of growth period.

Growth period	2022 Year	2023 Year
Germination period	4.29–5.11	5.8–5.18
Seedling period	5.12–5.27	5.19–6.4
Vine growth period	5.28–6.20	6.5–6.20
Flowering period	6.21–7.12	6.21–7.13
Maturity period	7.13–8.11	7.14–8.14

### 2.4 Soil sample collection and physicochemical properties determination

#### 2.4.1 Soil sample collection

In 2022 and 2023, soil samples were collected during the germination period (G), seedling stage (SE), vine extension period (SP), flowering stage (F), and maturity period (M) of pumpkins. For each organic fertilizer treatment plot, soil samples were collected from the 0–100 cm soil layer at three locations with an “S” shaped distribution, stratified at 20 cm intervals. After removing rocks and plant residues, the remaining soil was stored in sterilized plastic containers to determinate soil physical and chemical properties.

#### 2.4.2 Determination of soil bulk density

The soil bulk density is determined by the ring knife method. A representative undisturbed soil sample is cut with a ring knife of a certain volume, filled with soil, and the mass of dried soil (at 105°C) per unit volume is calculated as the bulk density of the soil, as shown in the formula below (Zhang et al., 2020):


(1) $$\gamma =\left(m_{2}-m_{1}\right)/V$$

Where, γ is bulk density of soil, g/cm^3^; m_1_ is mass of the ring knife, g; m_2_ is mass of ring knife plus dried soil, g; V is volume of the ring knife, cm^3^.

#### 2.4.3 Determination of soil nutrients

Soil organic carbon was determined by potassium dichromate oxidation–spectrophotometric method, using soil organic carbon analyzer of LH–SOC 350 (Beijing Lianhua Technology, ChN) (using the M–02 calibration curve with a value of 10.80). Available phosphorus in soil was determined by sodium bicarbonate extraction–molybdenum antimony anti–colorimetric method. Available potassium in soil was determined by ammonium acetate extraction–flame photometry method. Soil nitrate nitrogen content was determined by ultraviolet spectrophotometric colorimetric method.

### 2.5 Growth characteristics analysis method

#### 2.5.1 Logistic curve fitting of pumpkin growth dynamics

Logistic equation was used to fit dynamic changes of pumpkin growth indicators during the reproductive period, with the equation as (Tung et al., 2019):


(2) $$y=k/\left[1+a\cdot e^{-bt}\right]$$

Where, y is stem diameter (mm), vine length (cm), leaf area (cm^2^) of pumpkin at the moment time t; t is number of days after emergence, d; k is theoretical maximum growth value; a, b are both constants.

By differentiating the logistic equation, the start time of pumpkin rapid growth (T_1_, in days), the end time of rapid growth (T_2_, in days), the time to reach the fastest growth rate (T_0_, in days), and the fastest growth rate [V_max_, mm/cm/cm^2^/d] can be obtained, with the calculation formulas for each parameter as follows (Tung et al., 2019):


(3) $$T_{1}=\ln\left[a\left(2-\sqrt{3}\right)\right]/b$$

(4) $$T_{2}=\ln\left[a\left(2+\sqrt{3}\right)\right]/b$$

(5) $$V_{max}=kb/4$$

(6) $$T_{0}=lna/b$$

(7) $$V_{T}=\Delta y-\Delta T=\left(y_{2}-y_{1}\right)/\left(T_{2}-T_{1}\right)$$

Where, V_T_ represents the period when 65% cumulative growth occurs, defined as the rapid accumulation period, starting at T_1_ and ending at T_2_; other symbols as above.

#### 2.5.2 Water use efficiency

The water use efficiency is calculated as (Fernández et al., 2020):


(8) WUE=Y/ET

Where, WUE is water use efficiency, kg·ha^-1^·mm^-1^; Y is economic yield of pumpkin, kg·ha^-1^; ET is water consumption during the pumpkin growth period, mm.

Among them, the water consumption during the pumpkin growth period is calculated based on the principle of soil water balance in field, with the formula as follows (Zong et al., 2021):


(9) $$ET=W_{0}-W_{t}+W_{T}+P_{0}+K+M$$

Where, W_0_ and Wt are the initial and final water storage in the planned wet soil layer during time period t, respectively, mm; W_T_ is the water amount added as the depth of the planned wet soil layer increases, mm, with W_T_ = 0 in this experiment due to the depth of the planned wet soil layer remaining constant; P_0_ is the effective precipitation, mm; K is the supply amount of shallow buried groundwater to the planned wet soil layer, mm, with K = 0, that is not considered, due to the groundwater level in the experimental area below 20m; M is the irrigation water amount during time period t, mm.

#### 2.5.3 Yield and fertilizer partial factor productivity

Yield measurement at harvest: After pumpkins mature, pumpkin at each plot is harvested individually to determine pumpkin yield and its composition factors. Calculation formula for fertilizer partial factor productivity is (Ierna et al., 2011):


(10) PFP=Y/F

Where, PFP is the fertilizer partial factor productivity, kg·kg^-1^; F is the total amount of fertilizer applied, kg·ha^-1^.

### 2.6 Data analysis

All data were analyzed using Excel (2021) (Microsoft, USA) and Origin 2017 (OriginLab Corp., USA). One-way analysis of variance (ANOVA) was conducted by SPSS 25.0 (IBM, USA). Mantel correlation analysis of soil nutrients and yield was performed using the linkET package in R language version 4.3.1.

## 3 Results and analysis

### 3.1 Effects of organic fertilizer on pumpkin growth and development dynamics

**Figure 2:**
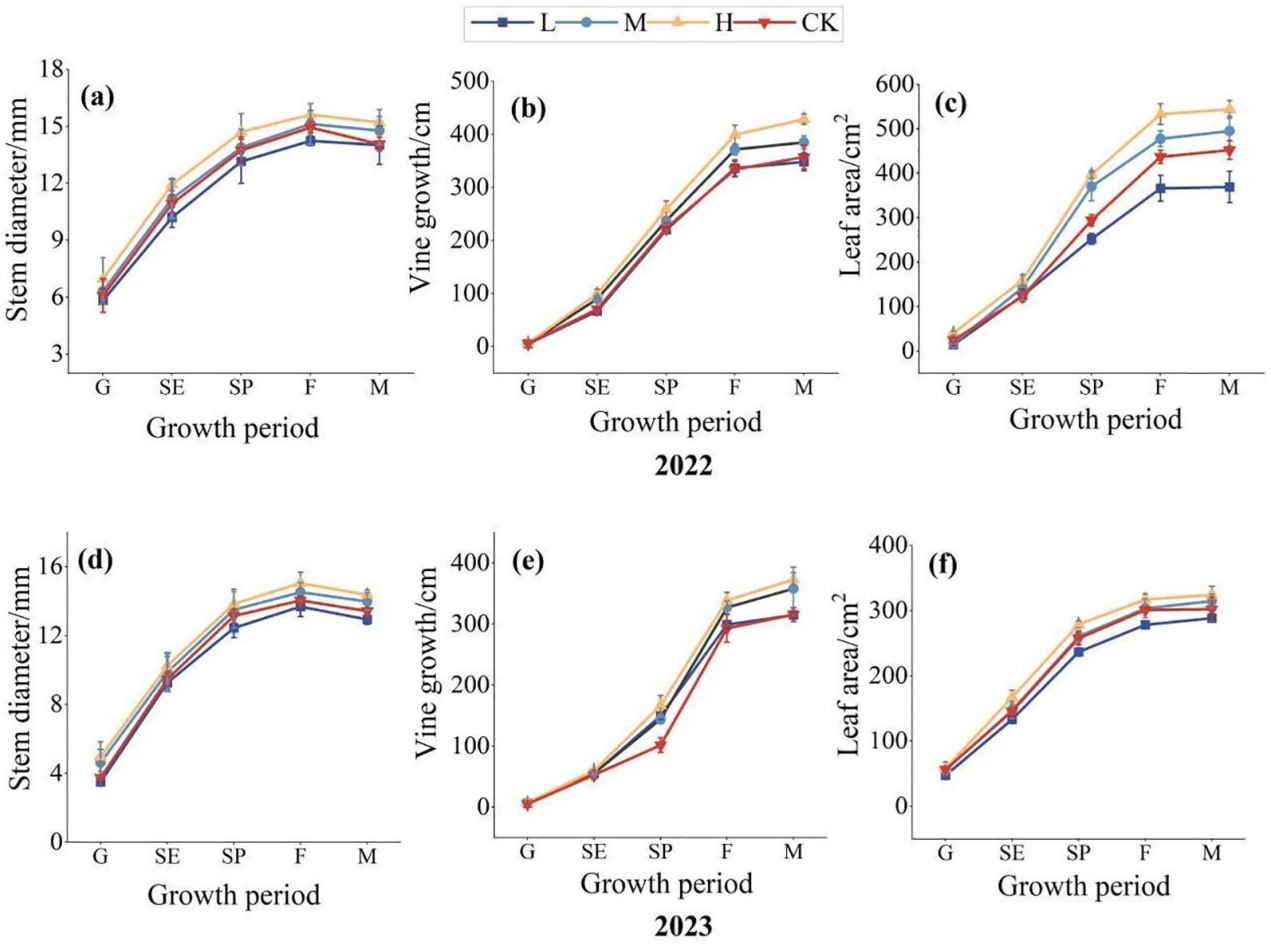
The influence of organic fertilizer on stem diameter, vine length, and leaf area during the growth period of pumpkins in 2022 and 2023. **(A–C)** represents the changes of stem diameter, vine length and leaf area in the growth period of pumpkin under different treatments in 2022; **(D–F)** represents the changes of stem diameter, vine length and leaf area in the growth period of pumpkin under different treatments in 2023.

The application of organic fertilizer had a significant dynamic effects on the growth and development of pumpkin (P<0.05), as illustrated in **Figure 2**. The data indicated that dynamic changes of pumpkin growth were similar over the two years from 2022 to 2023. Taking 2022 as an example, throughout the entire growth process of pumpkins, the trends on stem thickness for each treatment were consistent, as shown in **Figure 2A**. It was mainly characterized by rapid increase of the stem thickness in the early growth stage, which reached a peak and then slightly decreased due to the continuous loss of stem moisture as the pumpkin gradually matured. Among them, the stem thickness of pumpkins under H treatment was the largest, at 12.89 mm. **Figure 2B** reflected that vine length showed a stable upward trend during the entire growth period. The level of organic fertilizer application had a significant impact on the vine length of pumpkins (P<0.05), with the sizes of vine length in each treatment being H > M > CK > L. Among them, the H treatment had the largest value of 428.67 cm, representing an increase of 22.10%, 9.45%, and 20.31% compared to L, M, and CK, respectively. The pumpkin leaf area showed a gradual increasing trend at different levels of organic fertilizer application, with a relatively large growth rate from the germination period to the vine elongation period, as shown in **Figure 2C**. The sizes of leaf areas among the treatments also followed the order of H > M > CK > L. The leaf area under the high-level organic fertilizer treatment was the largest, with an increase of 25.81% compared to the control.

The logistic equation was used to fit the growth dynamics process of pumpkins from 2022 to 2023. The results showed that pumpkins growth throughout the entire growth period conformed to an “S”– shaped growth curve (**Table 4**). The trends between the maximum relative growth rate and average growth rate of pumpkins for each treatment were consistent. Compared to the control group (CK), the maximum relative growth rates of vine length and leaf area of pumpkins under H treatment increased by an average of 14.12% and 22.56% over two years. In 2022, compared to the CK, the average growth rates of stem diameter, vine length, and leaf area of pumpkins under H treatment increased by 3.96%, 10.31%, and 24.15%, respectively. The rapid growth duration of pumpkin stem diameter and vine length was extended by 1.91% and 9.41%, respectively, while the rapid growth duration of leaf area was shortened by 7.63%. In 2023, under the H treatment, the rapid growth duration of pumpkin stem diameter and vine length increased by 18.66% and 27.35% respectively, compared to the CK treatment. Furthermore, the maximum relative growth rates of stem diameter, vine length, and leaf area of pumpkins occurred 11.95%, 8.58%, and 6.88% earlier than CK.

**Table 4:** The logistic equation regression analysis of pumpkin growth dynamics under organic fertilizer treatment.

Year	Indicator	Treatment	Logistic equation	R^2^	V_max_ (mm/cm/cm^2^·d^-1^)	T_0_ (d)	T_1_ (d)	T_2_ (d)	△T (d)	V_T_
2022	Stem diameter/mm	L	*y*=14.125/[1+2.392·e^(-0.106^ *^t^* ^)^]	0.995**	0.374	8.238	-4.202	20.677	24.879	0.328
		M	*y*=14.876/[1+2.338·e^(-0.113^ *^t^* ^)^]	0.990**	0.419	7.545	-4.154	19.244	23.398	0.368
		H	*y*=15.387/(1+2.164·e^(-0.117t)^]	0.995**	0.452	6.576	-4.640	17.792	22.432	0.395
		CK	y=14.453/[1+2.498·e^(-0.12t)^]	0.981**	0.432	7.649	-3.357	18.655	22.012	0.380
	Vine length/cm	L	*y*=353.141/[1+39.116·e^(-0.121^ *^t^* ^)^]	0.994**	10.677	30.317	19.428	41.206	21.779	9.365
		M	*y*=392.539/[1+27.561·e^(-0.11^ *^t^* ^)^]	0.986**	10.789	30.166	18.187	42.144	23.958	3.818
		H	*y*=433.295/(1+25.211·e^(-0.106t)^]	0.988**	11.442	30.553	18.085	43.020	24.935	10.059
		CK	y=358.972/[1+33.351·e^(-0.116t)^]	0.995**	10.372	30.346	18.951	41.741	22.791	9.119
	Leaf area/cm^2^	L	*y*=331.802/[1+10.49·e^(-0.101^ *^t^* ^)^]	0.970**	9.711	26.369	13.593	39.145	25.552	7.260
		M	*y*=404.149/[1+13.245·e^(-0.112^ *^t^* ^)^]	0.993**	15.056	25.482	14.667	36.296	21.628	9.980
		H	*y*=437.081/(1+11.156·e^(-0.107t)^]	0.996**	15.294	25.810	13.951	37.669	23.718	10.126
		CK	y=382.526/[1+11.185·e^(-0.099t)^]	0.990**	11.882	28.407	15.643	41.170	25.527	8.156
2023	Stem diameter/mm	L	*y*=13.173/[1+5.755·e^(-0.152^ *^t^* ^)^]	0.986**	0.500	11.525	2.852	20.198	17.346	0.438
		M	*y*=14.231/[1+3.934·e^(-0.128^ *^t^* ^)^]	0.994**	0.456	10.681	0.411	20.950	20.539	0.400
		H	*y*=14.678/(1+3.612·e^(-0.124t)^]	0.990**	0.455	10.349	-0.264	20.963	21.228	0.399
		CK	y=13.696/[1+5.506·e^(-0.147t)^]	0.994**	0.504	11.586	2.641	20.531	17.890	0.442
	Vine length/cm	L	*y*=324.74/[1+51.694·e^(-0.113^ *^t^* ^)^]	0.979**	9.167	34.939	23.277	46.602	23.326	8.038
		M	*y*=368.04/[1+75.661·e^(-0.116^ *^t^* ^)^]	0.978**	10.676	37.286	25.936	48.636	22.700	9.361
		H	*y*=382.096/(1+51.991·e^(-0.11t)^]	0.986**	10.463	36.073	24.049	48.097	24.047	9.174
		CK	y=324.887/[1+235.976·e^(-0.139t)^]	0.950*	11.330	39.169	29.728	48.611	18.882	9.934
	Leaf area/cm^2^	L	*y*=286.99/[1+7.392·e^(-0.104^ *^t^* ^)^]	0.996**	7.494	19.152	6.543	31.760	25.217	6.571
		M	*y*=313.046/[1+7.043·e^(-0.104^ *^t^* ^)^]	0.997**	8.147	18.751	6.100	31.402	25.302	7.143
		H	*y*=321.417/(1+7.16·e^(-0.117t)^]	0.995**	9.303	17.002	5.628	28.377	22.749	8.157
		CK	y=304.236/[1+7.098·e^(-0.108t)^]	0.997**	8.203	18.171	5.960	30.382	24.422	7.192
Average	Stem diameter/mm	L	*y*=13.644/[1+3.555·e^(-0.126^ *^t^* ^)^]	0.991**	0.431	10.040	-0.384	20.467	20.851	0.377
		M	*y*=14.556/[1+2.991·e^(-0.12^ *^t^* ^)^]	0.993**	0.435	9.160	-1.849	20.161	22.010	0.383
		H	*y*=15.033/(1+2.752·e^(-0.12t)^]	0.993**	0.450	8.470	-2.548	19.480	22.028	0.395
		CK	y=14.083/[1+3.576·e^(-0.131t)^]	0.989**	0.462	9.720	-0.326	19.756	20.082	0.409
	Vine length/cm	L	*y*=339.231/[1+40.862·e^(-0.114^ *^t^* ^)^]	0.99**	9.681	32.503	20.966	44.040	23.074	8.480
		M	*y*=381.421/[1+36.447·e^(-0.107^ *^t^* ^)^]	0.983**	10.185	33.666	21.336	45.996	24.660	8.940
		H	*y*=408.563/(1+31.73·e^(-0.104t)^]	0.988**	10.636	33.201	20.554	45.849	25.295	9.320
		CK	y=345.623/[1+42.17·e^(-0.108t)^]	0.982**	9.320	34.690	22.481	46.900	24.420	8.180
	Leaf area/cm^2^	L	*y*=331.802/[1+10.49·e^(-0.101^ *^t^* ^)^]	0.983**	8.400	23.212	10.206	36.218	26.011	7.350
		M	*y*=404.149/[1+13.245·e^(-0.112^ *^t^* ^)^]	0.996**	11.343	23.013	11.282	34.743	23.461	9.930
		H	*y*=437.081/(1+11.156·e^(-0.107t)^]	0.996**	11.653	22.618	10.269	34.968	24.699	10.240
		CK	y=382.526/[1+11.185·e^(-0.099t)^]	0.992**	9.508	24.287	11.041	37.533	26.493	8.310

V_max_ is the maximum of relative growth rate, V_mean_ is average growth rate, T_0_ is the occurrence time of maximum relative growth rate, T_1_ is the start time of rapid growth, T_2_ is the end time of rapid growth, △T is rapid growth duration, T is the growth days. Different lowercase letters indicate significant differences between different processes(*, P < 0.05;**, P < 0.01).

### 3.2 Impact of organic fertilizer on soil bulk density

**Figure 3:**
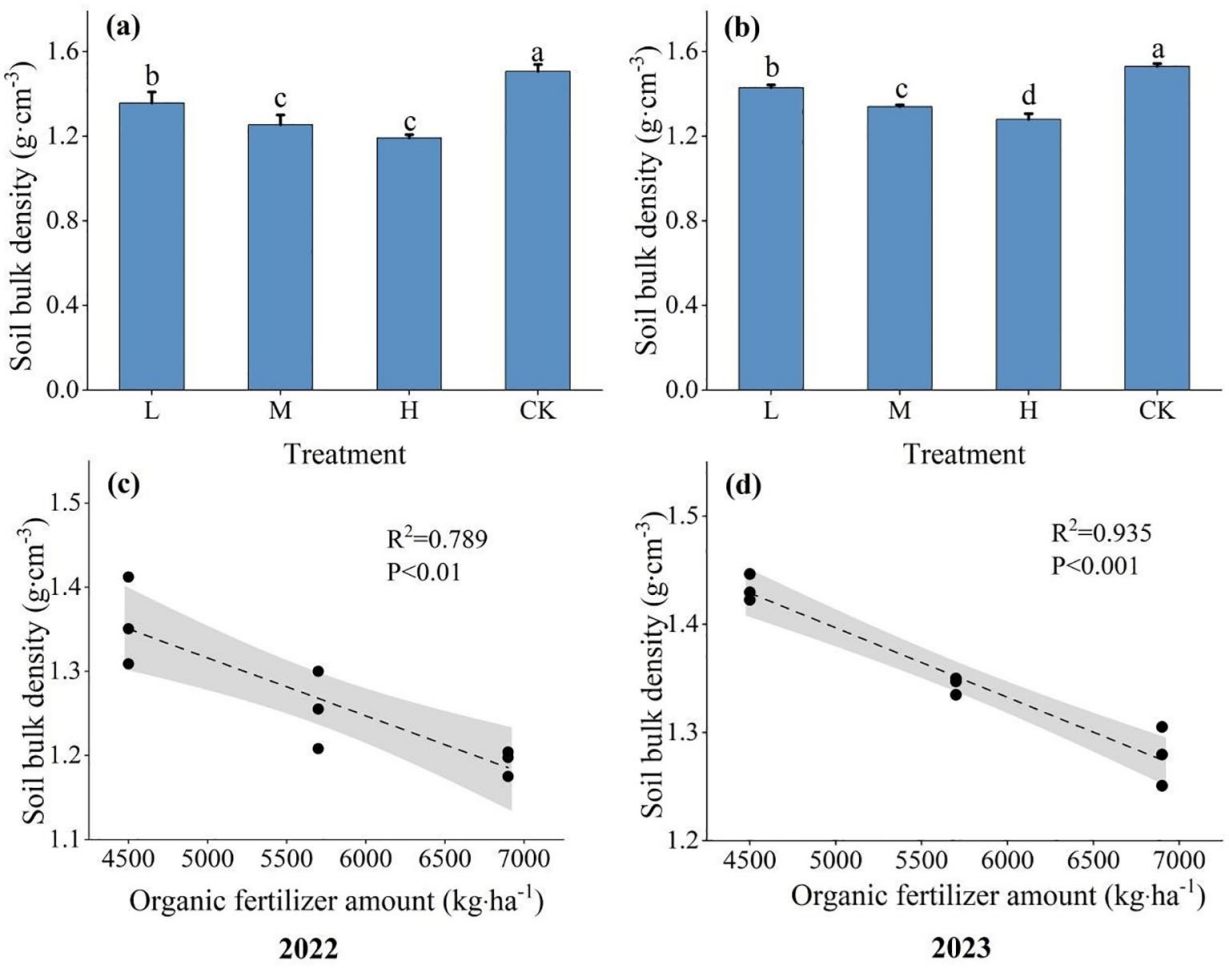
Changes on soil bulk density under different levels in different treatment. **(A)** represents the change of soil bulk density under different treatments; **(B)** represents the relationship between organic fertilizer and soil bulk density; **(C)** the relationship between different organic fertilizer contents and soil bulk density in 2022; **(D)** indicates the relationship between different organic fertilizer content and soil bulk density in 2023. Different lowercase letters indicate significant differences between treatments at the P <0.05 level.

After the application of organic fertilizer, a significant alteration in soil bulk density was observed, exhibiting a consistent pattern of change over the course of two years (**Figure 3**; **Supplementary Table 1**). In 2022, the application of organic fertilizer significantly reduced soil bulk density by 10.00–20.67% compared to the CK (P<0.05). With the growing utilization of organic fertilizer, there has been a reduction in soil bulk density, indicating a significant negative correlation between the two. The order of soil bulk density among different treatments was CK > L > M > H, among which there were significant differences between H, M, L and CK (P<0.05), but no significant difference between H and M treatments (P>0.05). In 2023, compared to CK, the application of organic fertilizer significantly reduced soil bulk density by 6.54–16.34%, and there was a significant negative correlation between application amount of organic fertilizer and soil bulk density. It was apparent that the utilization of organic fertilizer could significantly improve soil bulk density.

### 3.3 Effects of organic fertilizer on soil nutrients

#### 3.3.1 Effects of organic fertilizer on soil organic carbon

**Figure 4:**
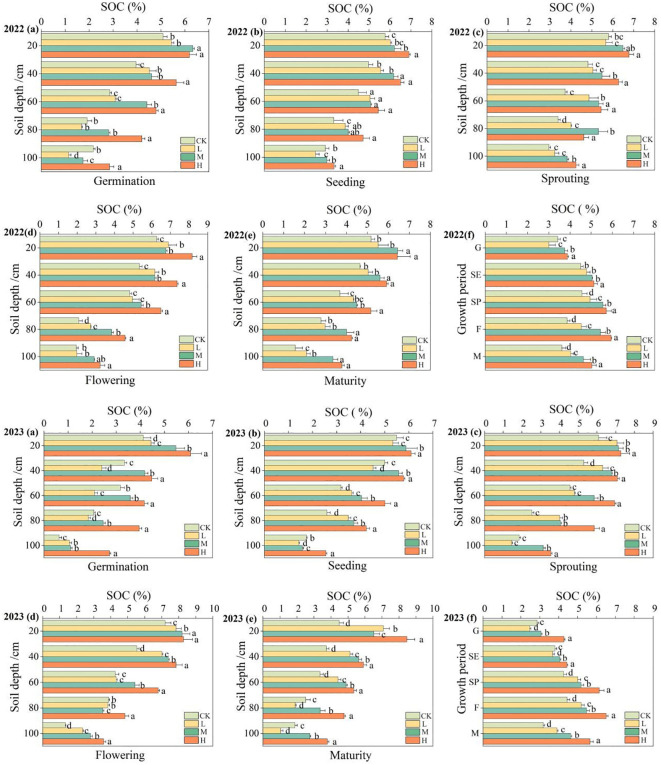
Changes on soil organic carbon content at 0 ‒ 100cm soil layers in different periods. The figures **(A–F)** represent the changes on soil organic carbon content during the germination period, seedling period, vine extension period, flowering period, maturation period, and full growth period, respectively; SOC represents soil organic carbon; different lowercase letters in the figure indicate significant differences between treatments in the same group (P <0.05), the same below.

**Figure 4** and **Supplementary Table 2** illustrates the impact of applying organic fertilizer on soil organic carbon. The data indicated that in both 2022 and 2023, there was a consistent trend of decreasing organic carbon content in farmland soil as the depth increased. The organic carbon content in the surface soil was significantly higher than that in the subsoil, and the rate of decrease in organic carbon content increased with the increase of soil depth. Under the H treatment, the soil organic carbon content was significantly higher than that under CK treatment (P<0.05). It also gradually increased with the increase of organic fertilizer application amount under different treatments. Taking the maturity stage in 2022 as an example, the rate of reduction in organic carbon content gradually increased with the increase of soil depth, with the decreasing by 11.19%, 20.50%, 27.77%, and 38.16% at each 20 cm interval in 0–100 cm soil layer, respectively. The application of organic fertilizer significantly increased soil organic carbon by 11.83–43.05% compared to CK. In the entire growth period of pumpkins, the soil organic carbon content exhibited a pattern of increasing first and then decreasing, with the order of H > M > L > CK among different treatments. Compared to the germination stage, the soil organic carbon content at the maturity stage increased by 29.62% with H treatment, 23.64% with M treatment, 34.33% with L treatment, while only 5.67% with CK treatment.

#### 3.3.2 Effects of organic fertilizer on soil available phosphorus

**Figure 5:**
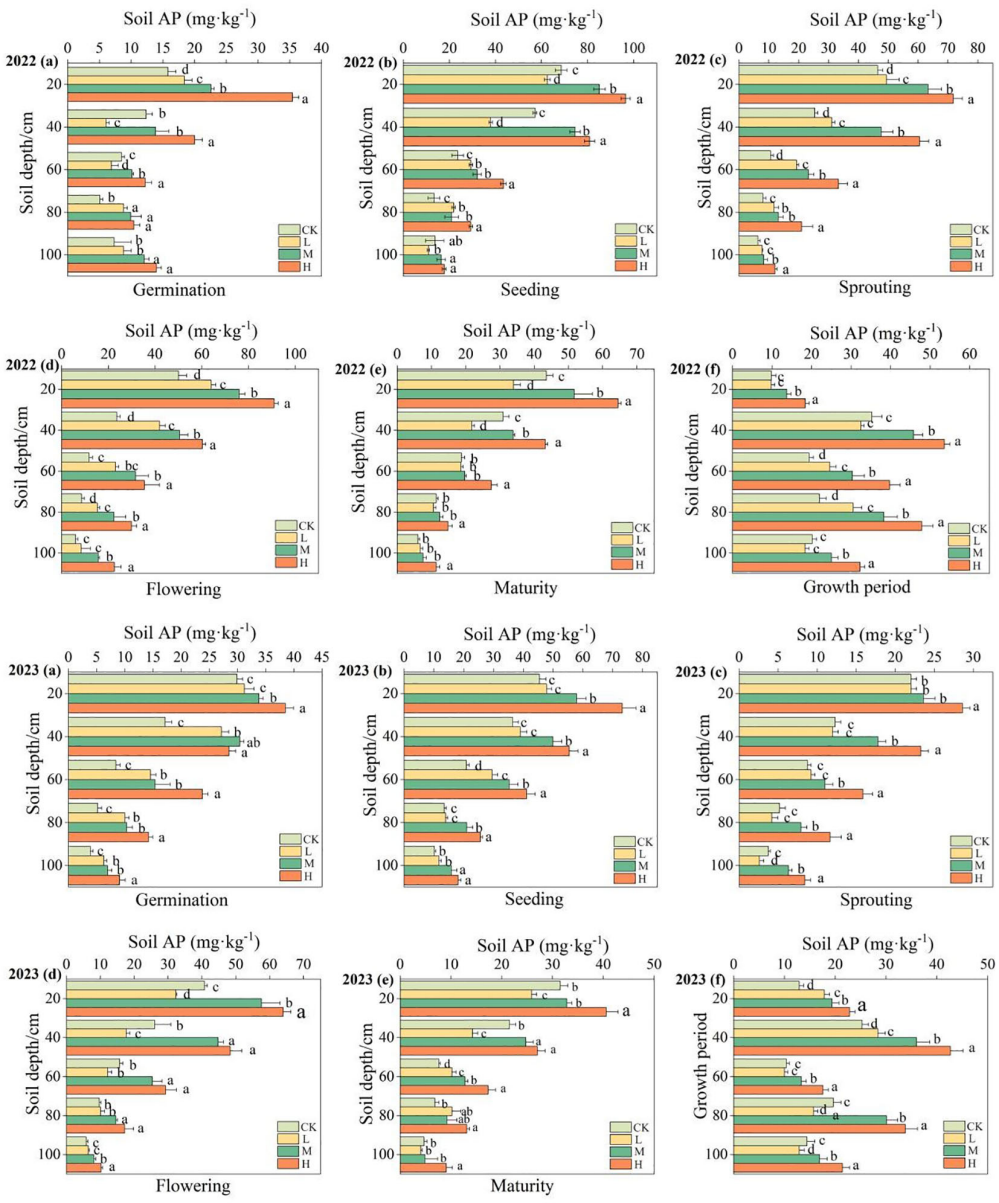
Changes on available phosphorus content at 0 ‒ 100cm soil layers in different periods. AP in the figure represents available phosphorus. **(A–F)** represent the changes on available phosphorus content during the germination period, seedling period, vine extension period, flowering period, maturation period, and full growth period.

The changes in available phosphorus content of soil profile during each period under various fertilizer treatments are illustrated in **Figure 5** and **Supplementary Table 3**. In 2022 and 2023, the effects of different fertilization levels on soil profile available phosphorus content were similar, showing a gradual decrease in available phosphorus content with the increasing of soil depth. The decrease rate of available phosphorus content in the 0–60 cm soil layer was significantly higher than that in the 60–100 cm soil layer. It could be observed that the soil available phosphorus content gradually increased with the increase of the organic fertilizers application amount. It under the H treatment was significantly higher than that under the CK treatment (P<0.05). The available phosphorus content in the 0–40 cm soil layer was significantly higher than in the 40–60 cm soil layer, with the soil available phosphorus content in the 0–40 cm soil layer among different treatments showing H > M > L > CK. The soil available phosphorus content during the entire growth period showed an M-shaped variation pattern. Compared to the CK treatment, the available phosphorus content significantly increased by 74.93% under the H level, 30.27% under the M level.

#### 3.3.3 Effect of organic fertilizer on soil available potassium

**Figure 6:**
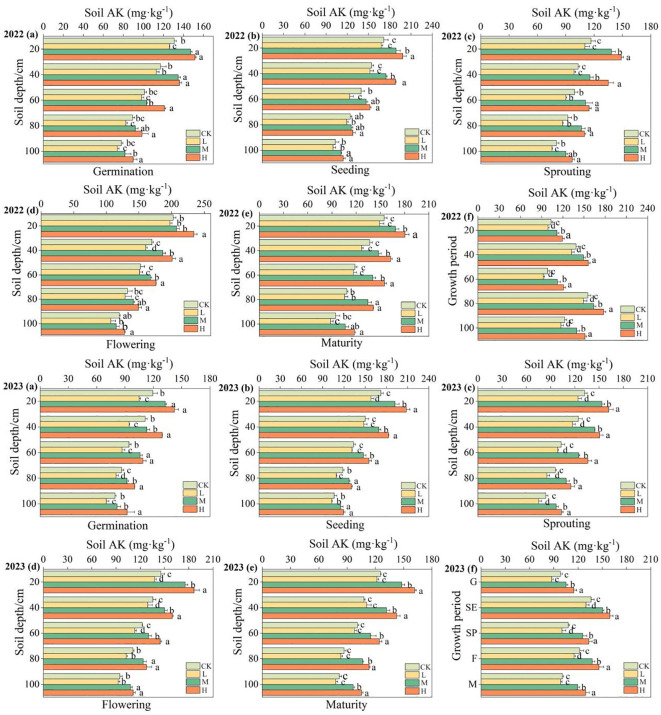
Changes on available potassium content at 0 - 100cm soil layers in different periods. AK in the figure represents available potassium. **(A–F)** represent the changes on available potassium content during the germination period, seedling period, vine extension period, flowering period, maturation period, and full growth period.

The impact of organic fertilizer on soil’s available potassium content during the entire growth period of pumpkins in 2022 and 2023 was similar (**Figure 6**; **Supplementary Table 4**). The soil available potassium content gradually decreased with the increase of soil depth, increased with the increase of organic fertilizer application amount. The change rate of available potassium content is the highest in the surface soil (0–40 cm), with significantly higher levels under the H and M treatments than that under the control treatment (P<0.05). In the year 2022, for example, the available potassium content increased by 18.00% under H treatment, 9.90% under M treatment, compared to the CK. Throughout the growth period, there were two peaks of the available potassium content, occurring at the seedling stage and flowering stage, with the maximum value at the flowering stage. In particular, the available potassium content reaches its peak at 179.89 mg·kg^-1^ under the H treatment.

#### 3.3.4 Effects of organic fertilizer on soil nitrate nitrogen

**Figure 7:**
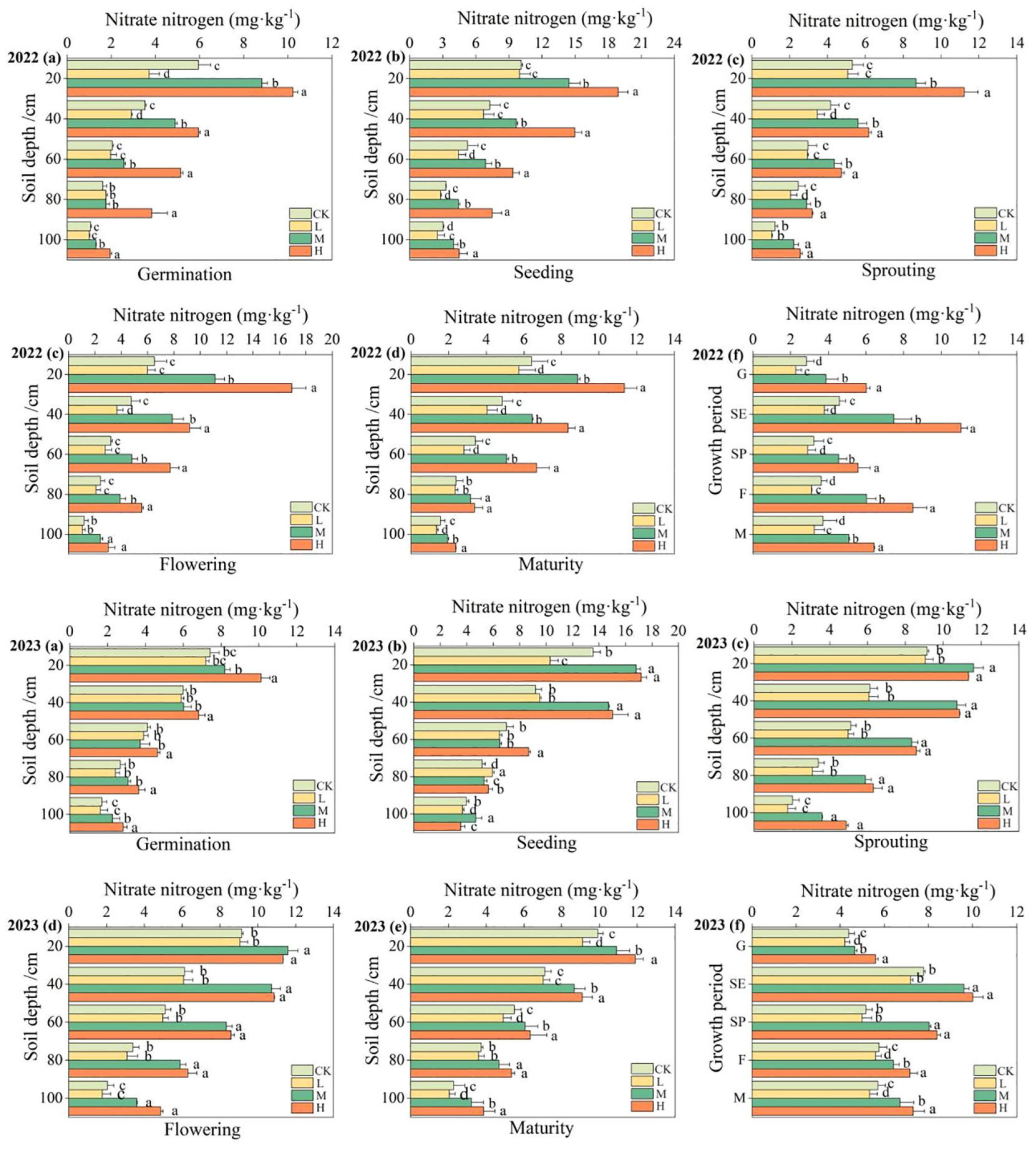
Changes on soil nitrate nitrogen content at 0 - 100cm soil layers in different periods. **(A–F)** represent the changes on soil nitrate nitrogen content during the germination period, seedling period, vine extension period, flowering period, maturation period, and full growth period.

The impact of organic fertilizer on soil nitrate nitrogen content during the entire growth period of pumpkins in 2022 and 2023 is illustrated in **Figure 7** and **Supplementary Table 5**. With the increase of soil depth, the soil nitrate nitrogen content gradually decreased, and the change rate at the 0–20cm soil layer was obviously larger. The changing patterns at different pumpkin growth stages were similar, with the trend of soil nitrate nitrogen content increasing with the increase of fertilizer application. Taking 2022 as an example, compared to the CK, the nitrate nitrogen content increased by 95.17% in the H treatment, and 36.47% in the M treatment, while decreased by 13.05% in the L treatment. During the whole growth period of pumpkins, soil nitrate nitrogen content showed a pattern of increase followed by decrease, with its maximum value at the seedling stage. The soil nitrate nitrogen content at maturity significantly decreased compared to the germination stage, with a decrease of 36.91% in the H treatment, 18.00% in the M treatment, and 58.99% in the L treatment, while an increase of 30.45% in the CK treatment.

### 3.4 Effect of organic fertilizer on pumpkin yield and water-fertilizer use efficiency

The provision of adequate nutrients is essential to ensure high crop yield, as well as to facilitate the growth and development of crops and improve the efficiency of fertilizer utilization. The effect of organic fertilizer on pumpkin yield in 2022 and 2023 was significant (P<0.05) (**Table 5**). The data indicated that pumpkin yield increased with the application of organic fertilizer within a certain extent. From individual fruit weight and final yield, the yield under different fertilization conditions was ranked as M > H > L > CK. Compared to the CK, the application of organic fertilizer increased pumpkin yield by 4.54–25.97%, improved water use efficiency by 2.21–19.24% on average in 2022 and 2023. Among them, pumpkin yield, individual fruit weight, and water use efficiency reached their maximum values of 31,101.13 kg·ha^-1^, 1.51 kg, and 69.24 kg·ha^-1^·mm^-1^ under the M treatment respectively, significantly higher than them under CK and low level treatment of organic fertilizer (P<0.05). Compared to CK, pumpkin yield, individual fruit weight, and water use efficiency increased an average by 6,412.07 kg·ha^-1^, 0.53 kg, and 11.17 kg·ha^-1^·mm^-1^, respectively, over two years. The M and H treatments significantly improved pumpkin yield, individual fruit weight, and water use efficiency, with no significant difference between them (P>0.05). As the amount of organic fertilizer applied increased, the fertilizer partial productivity gradually decreased, with the maximum fertilizer partial productivity (PFP) under CK treatment. Compared to L, PFP of M treatment was decreased by 5.12%, and that of H treatment was decreased by 49.79%.

**Table 5:** Effects of organic fertilizer on pumpkin yield and water use efficiency.

Year	Treatment	Single melon weight (kg)	Yield (kg·ha^-1^)	Water use efficiency (kg·ha^-1^·mm^-1^)	Fertilizer partial productivity
2022	L	0.82c	24914.16c	51.51b	5.54b
	M	1.60a	30726.13a	62.94a	5.39c
	H	1.41a	26044.72b	54.01b	3.77d
	CK	1.08b	24119.46d	52.51b	17.87a
2023	L	1.01bc	26705.73b	65.51b	5.93b
	M	1.42a	31126.22a	75.48a	5.52b
	H	1.21ab	26794.72b	65.65b	3.88c
	CK	0.89c	25258.67b	64.59b	18.71a
Average	L	0.92b	25809.94b	57.91b	5.74b
	M	1.51a	30926.18a	68.21a	5.46b
	H	1.31a	26419.72b	59.34b	3.83c
	CK	0.99b	24689.06c	58.06b	18.29a

Different lowercase letters indicate significant differences between treatments at the *P* <0.05 level.

### 3.5 Correlation analysis of soil nutrients with pumpkin growth dynamics and yield

**Figure 8:**
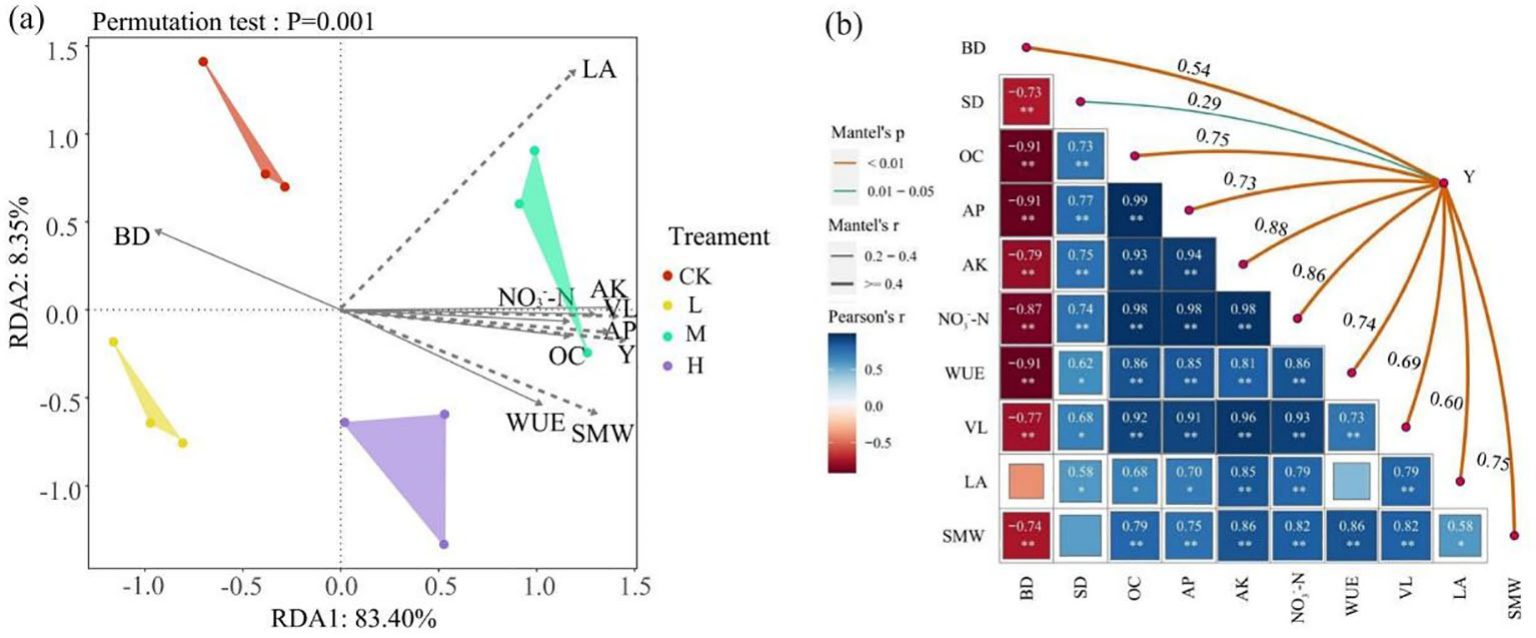
The impact of soil nutrients on pumpkin yield. **(A)** Redundancy analysis (RDA) results between soil factors and pumpkin traits, **(B)** Mantel test results between soil factors and pumpkin traits. BD, SD, OC, AP, AK, NO_3_^-^-N, WUE, VL, LA, SMW, and Y represent soil bulk density, stem diameter, soil organic carbon, soil available phosphorus, soil available potassium, soil nitrate nitrogen, crop water use efficiency, vine length, leaf area, single fruit weight, and yield, respectively. The larger the square, the stronger the correlation coefficient; the smaller the square, the weaker the correlation coefficient. Blue indicates positive correlation, red indicates negative correlation, and darker color indicates stronger correlation. * indicates P<0.05, ** indicates P<0.01.

The redundant analysis (RDA) and Mantel test results of soil nutrient, pumpkin growth dynamics, and yield in 2022 and 2023 (**Figures 8A, B**) show that soil bulk density is significantly negatively correlated with pumpkin stem thickness, vine length, and yield (P<0.01), while there is no significant correlation with pumpkin leaf area (P>0.05). Soil organic carbon, available phosphorus, available potassium, nitrate nitrogen, and water use efficiency are all significantly positively correlated with pumpkin yield (P<0.01).

## 4 Discussions

### 4.1 Impact of organic fertilizer on soil bulk density and nutrients

This founding of this study indicated that the application of organic fertilizer can significantly improve soil bulk density. With the increasing application of organic fertilizer, there was a gradual decrease in soil bulk, indicating a significant negative correlation. Some scholars had got the same conclusion: the application of organic fertilizer could increase soil porosity, reduce bulk density, and improve water holding capacity. Different application amounts of organic fertilizer had different effects on regulating balance of crops nutrient absorption and improving soil structure (Sunny et al., 2022; Li et al., 2021a; Gul-Lalay et al., 2024).

The organic fertilizers are distinguished by their wide range of nutrients and prolonged effectiveness. This study has demonstrated that the application of organic fertilizer can enhance soil fertility and improve both the physical and chemical properties of farmland soil. It significantly increased organic carbon, alkali-hydrolyzable nitrogen, available phosphorus, and available potassium content compared to the control treatment. There were two times increase significant increase process in the whole growth period, which were mainly related to two times of topdressing fertilization. The contents of available potassium, available phosphorus, and nitrate nitrogen in the soil were all increased in each time of topdressing fertilization period. Similarly, it had been found that long-term application of organic fertilizers could also increase the accumulation and supply capacity of soil nutrients, as well as significantly increase the storage of soil carbon and nitrogen, with a long validity period (Gai et al., 2018). Whereas long-term application of chemical fertilizers could also increase the content of available potassium, but the effect is not as obvious as that of applying organic fertilizers (Fan et al., 2020). Organic nitrogen within organic fertilizers was slowly mineralized and released, although it could not quickly supply a large amount of nutrients in a timely at the critical crop growth periods, its effectiveness was more long-lasting than that of inorganic fertilizer (Li et al., 2020b). Studies had found that organic fertilizers significantly increase soil organic carbon by 15.6% and improve available nutrients by 2.3% to 20.2% (Liu et al., 2021b). Results also indicated that applying organic fertilizers could significantly increase soil organic matter, total nitrogen, available potassium, and available phosphorus content, but had no significant impact on nitrate nitrogen and ammonium nitrogen content, compared with 100% application of chemical fertilizer (Fan et al., 2023).

This study also indicated that soil organic carbon, available phosphorus, available potassium, and nitrate nitrogen content gradually increased with the increase of organic fertilizer application, but the changes of the 1st year and 2nd year were not significant. Similar conclusions had been drawn by Shi et al., who found that soil organic carbon, alkaline nitrogen, available phosphorus, and available potassium content all showed a gradual increase with the increase of organic fertilizer application, and the increase of various indicators in the 2nd year was more obvious (Shi et al., 2017). It was related to the fact that organic fertilizer decomposed exogenous organic matter in the soil by increasing the activity of functional bacteria, thereby releasing nitrogen, phosphorus, and potassium, and activating insoluble nutrients such as nitrogen, phosphorus, and potassium in the soil (Shi et al., 2017). This study concluded that compared with the control group, the application of organic fertilizer could significantly increase soil organic carbon, alkaline nitrogen, available phosphorus, and available potassium content, improve soil nutrients, and enhance soil fertility. The reason for this is related to the fact of effective bacteria (Bacillus subtilis + Bacillus licheniformis) ≥ 0.5 billion/ml and amino acids ≥ 3% from the tested organic fertilizer. It was indicated that the addition of effective bacteria (Bacillus subtilis + Bacillus licheniformis) and amino acids in organic fertilizer could further increase soil nutrient content. Li also showed that long-term use of organic fertilizer could increase microbial biomass and enzyme activity, improve the quantity and quality of soil organic matter, and organic fertilizer added with Bacillus subtilis could further increase soil nutrient content (Li et al., 2021a). The improvement on soil fertility was mainly due to the reproduction of microorganisms in organic fertilizer, which could regulate the accumulation and cycling of soil nutrients (Li et al., 2021a). Organic fertilizer could activate soil nutrients and thus improve soil fertility. The biodegradation and transformation of organic matter in organic fertilizer could significantly increase the metabolic activity of microorganisms, activate soil nutrients, and promote nutrient absorption of crops (Wang et al., 2004; Shi et al., 2014; Nagaraju et al., 2012).

### 4.2 Effects of organic fertilizer on growth dynamics, yield, and water and fertilizer utilization efficiency of pumpkin

This study indicated that the application of organic fertilizer can enhance the growth and development of pumpkin, with an increase in vine length, stem thickness, and leaf area corresponding to higher application rates. Similar conclusions had been reported that the application of organic fertilizer with the phylum Ascomycota, could promote the nutrients absorption of plant roots system, thus plant growth and development (Toffa et al., 2021). Adebayo found that the application of organic fertilizer significantly increased vine length and yield of pumpkin (Olowoake and Adeyemo, 2020). Research also showed that the application of organic fertilizer had the most significant improvement effect on crop height and stem thickness, with the largest single plant leaf area (Wang et al., 2020b). The application of organic fertilizer could significantly increase the plant height, number of leaves, and leaf area of crops, having higher economic benefits than inorganic fertilizer (Mahamad et al., 2022).

The application of fertilizer is a key determinant of pumpkin yield and water utilization efficiency. This study also found that the application of organic fertilizer was beneficial to the formation of pumpkin yield. A large number of studies had shown that fertilizers, as the main source of crop nutrients, were directly participate in or regulate crop nutrient metabolism and cycling, and were closely related to crop yield (Gautam et al., 2022). Studies had shown that within a certain range of fertilization application amount, the application of organic fertilizer could promote to increase crop yield (Moritz et al., 2023; Vinh and Quang, 2022; Liu et al., 2023). In this study, it was also found that pumpkin yield increased to a certain extent with the increase in the application amount of organic fertilizer, that is, an appropriate increase in organic fertilizer could contribute to pumpkin yield formation. However, under the high-level organic fertilizer treatment, the pumpkin yield decreased. The data from 2022 and 2023 showed that compared to the high-level organic fertilizer treatment, the medium-level organic fertilizer treatment increased the pumpkin yield by 4681.41 kg·ha^-1^ and 4331.50 kg·ha^-1^, respectively. It was because that organic fertilizer had a long effective period, and high amount of organic fertilizer could lead to pumpkin stem lengthening at maturity, and yields were reduced instead. Li also found that the application of organic fertilizer could increase crop yield, but excessive application did not achieve the desired increase in yield and instead reduced economic income (Li et al., 2022). This was because excessive application of organic fertilizer was accompanied by an increase in the number of pathogenic microorganisms in the soil and overabundance of nutrient accumulation, which was also easy to be lost and not conducive to crop growth (Liu et al., 2020).

This study also revealed that overall water utilization efficiency of pumpkin was improved through the application of organic fertilizer, and the yield of the medium and high level organic fertilizer treatments was higher than that of the control treatment. It could be seen that the application of organic fertilizer also needed to be within an appropriate range to promote yield increase and improve water and fertilizer utilization efficiency (Xiang et al., 2022).

In addition, some studies had shown that the significant impact of bio-organic fertilizer and chemical fertilizer on soil lies in soil microbial community structure. Compared with no fertilizer or chemical fertilizer application, the application of organic fertilizer improved the resistance of soil microbial community to disturbance, indicating that organic fertilizer changes the structure of soil microbial community, thereby affecting crop yield (Fan et al., 2023; Cui et al., 2018; Francioli et al., 2016; Legrand et al., 2018). It was particularly important to reasonably use organic fertilizer for soil fertility improvement, in order to reduce nutrient loss and mitigate soil environmental pollution risks. Excessive application of organic fertilizer could also lead to the salts accumulation, heavy metals and a decrease in the effectiveness of certain elements (Wang et al., 2020a; Wang et al., 2010; Muhammad et al., 2020). Therefore, further research would be needed to investigate the specific effects mechanisms of organic fertilizer application on crop yield, microorganisms, and certain elements.

## 5 Conclusion

1. By analyzing the effects of organic fertilizer on soil nutrients, growth and yield of pumpkin, the results showed that high organic fertilizer treatment was more beneficial to the growth of stem thickness, vine length, and leaf area of pumpkin.
2. Compared to the CK, the application of various organic fertilizer treatments resulted in a significantly reduction in soil bulk density, and there was a significant negative correlation between the amount of organic fertilizer applied and soil bulk density.
3. The content of organic carbon, available potassium, available phosphorus, and nitrate nitrogen in the 0–100cm soil layer exhibited a gradual decline following application of various organic fertilizer treatments. Compared to the CK, the contents of organic carbon, available potassium, available phosphorus, and nitrate nitrogen were significantly increased by medium and high level of organic fertilizer treatment.
4. Under different treatments, the yield and water use efficiency of pumpkin treated with medium level organic fertilizer were the highest. Fertilizer partial productivity gradually decreased with the increase of organic fertilizer application amount. There were extremely significant positive correlations between pumpkin yield and stem thickness, vine length, organic carbon, available phosphorus, available potassium, nitrate nitrogen, single fruit weight, and water-fertilizer use efficiency.

In a comprehensive analysis of the effects of varying levels of organic fertilizer on pumpkin yield and soil nutrients content, the M treatment (application of 5700 kg·ha^-1^ organic fertilizer including base fertilizer 4800 kg·ha^-1^, 900 kg·ha^-1^ follow-up fertilizer) has proven to be advantageous for the cultivation of pumpkins in the arid regions of northwest China and similar areas.

## Data Availability

The original contributions presented in the study are included in the article/**Supplementary Material**. Further inquiries can be directed to the corresponding author.
