# Supplementary material for: Organic fertilizer increases pumpkin production by improving soil fertility
Source: Front Plant Sci. 2024 Nov 14;15:1467931. doi: 10.3389/fpls.2024.1467931 (PMC11603078; doi:10.3389/fpls.2024.1467931)
Supplement: Supplementary file 1 [file Table1.docx]

**Supporting Information**

**Table S1 Changes on soil bulk density under different levels in different treatment.**

| Treatment | Soil sulk density (g·cm^-3^) | |
| --- | --- | --- |
|  | 2022 | 2023 |
| L | 1.35±0.05b | 1.43±0.012b |
| M | 1.25±0.05c | 1.34±0.008c |
| H | 1.19±0.02c | 1.28±0.027d |
| CK | 1.50±0.03a | 1.53±0.013a |

**Table S2 Changes on soil organic carbon content at 0‒100cm soil layers in different periods.**

| year | Fertile period | | Soil depth (cm) | CK | L | | M | H | |
| --- | --- | --- | --- | --- | --- | --- | --- | --- | --- |
| 2022 | Germination | | 20 | 5.09±0.16b | | 5.43±0.10b | 6.33±0.06a | | 6.19±0.29a |
|  |  |  | 40 | 3.96±0.13c | | 4.51±0.28b | 4.61±0.25b | | 5.64±0.31a |
|  |  |  | 60 | 2.86±0.07c | | 3.09±0.04c | 4.41±0.20b | | 4.79±0.09a |
|  |  |  | 80 | 1.92±0.18b | | 1.67±0.05b | 2.82±0.05b | | 4.2±0.13a |
|  |  |  | 100 | 2.17±0.08b | | 1.14±0.10d | 1.75±0.18c | | 2.85±0.18a |
|  | Seeding | | 20 | 5.78±0.16c | | 6.02±0.06bc | 6.21±0.31b | | 6.91±0.06a |
|  |  |  | 40 | 4.98±0.20b | | 5.56±0.13b | 6.18±0.20a | | 6.51±0.16a |
|  |  |  | 60 | 4.49±0.38a | | 5.06±0.21a | 5.08±0.05a | | 5.45±0.29a |
|  |  |  | 80 | 3.32±0.43b | | 3.87±0.14ab | 4.01±0.06ab | | 4.72±0.30a |
|  |  |  | 100 | 2.92±0.16b | | 2.45±0.17c | 2.99±0.13b | | 3.33±0.07a |
|  | Sprouting | | 20 | 5.81±0.12bc | | 5.67±0.30c | 6.49±0.08ab | | 6.78±0.21a |
|  |  |  | 40 | 4.82±0.21c | | 5.05±0.17c | 5.49±0.37b | | 6.28±0.17a |
|  |  |  | 60 | 3.73±0.09c | | 4.87±0.43b | 5.33±0.21a | | 5.45±0.31a |
|  |  |  | 80 | 3.39±0.10d | | 4.01±0.02c | 5.32±0.43a | | 4.62±0.23b |
|  |  |  | 100 | 2.96±0.07c | | 3.22±0.21c | 3.83±0.07b | | 4.25±0.13a |
|  | Flowering | | 20 | 6.27±0.08c | | 6.9±0.44b | 6.76±0.06b | | 8.17±0.25a |
|  |  |  | 40 | 5.34±0.14c | | 6.17±0.22b | 6.16±0.07b | | 7.37±0.04a |
|  |  |  | 60 | 4.82±0.10c | | 4.96±0.36c | 5.43±0.12b | | 6.49±0.08a |
|  |  |  | 80 | 2.08±0.19d | | 2.72±0.02c | 3.85±0.09b | | 4.57±0.04a |
|  |  |  | 100 | 1.95±0.10b | | 1.98±0.27b | 2.91±0.02ab | | 3.23±0.25a |
|  | Maturity | | 20 | 5.18±0.16b | | 5.51±0.48b | 6.44±0.24a | | 6.43±0.61a |
|  |  |  | 40 | 4.64±0.02b | | 5.04±0.21b | 5.6±0.20a | | 5.91±0.08a |
|  |  |  | 60 | 3.68±0.40c | | 4.33±0.02bc | 4.48±0.05b | | 5.16±0.29a |
|  |  |  | 80 | 2.78±0.23b | | 2.98±0.24b | 4.01±0.34a | | 4.24±0.05a |
|  |  |  | 100 | 1.56±0.34c | | 2.09±0.18b | 3.35±0.23a | | 3.78±0.11a |
|  | Germination | | | 3.42±0.12c | | 3.00±0.31c | 3.75±0.14b | | 3.88±0.05a |
|  | Seeding | | | 4.51±0.11b | | 4.78±0.17b | 5.05±0.02b | | 5.13±0.17a |
|  | Sprouting | | | 4.57±0.24d | | 4.95±0.31c | 5.55±0.14b | | 5.72±0.23a |
|  | Flowering | | | 3.87±0.13d | | 4.54±0.23c | 5.44±0.23b | | 5.94±0.02a |
|  | Maturity | | | 3.62±0.18d | | 4.03±0.13c | 4.63±0.3b | | 5.03±0.21a |
| 2023 | Germination | 20 | | 4.12±0.31d | | 4.45±0.12c | 5.48±0.36b | | 6.09±0.44a |
|  |  | 40 | | 3.34±0.10c | | 2.41±0.17d | 4.18±0.14b | | 4.48±0.24a |
|  |  | 60 | | 3.17±0.17b | | 2.09±0.14c | 3.59±0.11b | | 4.17±0.15a |
|  |  | 80 | | 2.07±0.06c | | 1.84±0.09d | 2.46±0.09b | | 3.96±0.1a |
|  |  | 100 | | 0.63±0.10c | | 1.06±0.09b | 1.10±0.08b | | 2.72±0.05a |
|  | Seeding | 20 | | 5.50±0.27c | | 5.33±0.24c | 5.89±0.46b | | 6.10±0.16a |
|  |  | 40 | | 5.00±0.13c | | 4.52±0.12d | 5.58±0.15b | | 5.79±0.05a |
|  |  | 60 | | 3.18±0.06d | | 3.63±0.06c | 4.04±0.24b | | 5.01±0.24a |
|  |  | 80 | | 2.60±0.14d | | 3.49±0.11c | 3.72±0.16b | | 4.23±0.14a |
|  |  | 100 | | 1.75±0.03b | | 1.43±0.03d | 1.58±0.05c | | 2.55±0.04a |
|  | Sprouting | 20 | | 6.09±0.44c | | 7.07±0.35b | 7.15±0.24b | | 7.28±0.46a |
|  |  | 40 | | 5.3±0.21d | | 6.31±0.29c | 6.79±0.03b | | 7.09±0.10a |
|  |  | 60 | | 4.56±0.05c | | 4.79±0.05c | 5.85±0.20b | | 6.95±0.07a |
|  |  | 80 | | 2.53±0.11c | | 4.01±0.16b | 4.06±0.05b | | 5.86±0.27a |
|  |  | 100 | | 1.85±0.07c | | 1.45±0.04c | 3.14±0.10b | | 3.54±0.06a |
|  | Flowering | 20 | | 7.2±0.35c | | 7.85±0.3b | 8.2±0.46a | | 8.28±0.53a |
|  |  | 40 | | 5.54±0.13d | | 7.01±0.05c | 7.39±0.06b | | 7.85±0.35a |
|  |  | 60 | | 4.27±0.21c | | 4.33±0.06c | 5.43±0.24b | | 6.79±0.08a |
|  |  | 80 | | 3.87±0.06b | | 3.83±0.11b | 3.54±0.06c | | 4.84±0.22a |
|  |  | 100 | | 1.34±0.03d | | 2.35±0.04c | 2.82±0.11b | | 3.59±0.13a |
|  | Maturity | 20 | | 4.48±0.25d | | 7.08±0.36b | 6.52±0.34c | | 8.47±0.46a |
|  |  | 40 | | 3.73±0.16d | | 5.12±0.15c | 5.64±0.16b | | 5.92±0.18a |
|  |  | 60 | | 3.35±0.20d | | 4.42±0.16c | 4.89±0.07b | | 5.35±0.15a |
|  |  | 80 | | 2.52±0.27c | | 1.9±0.06d | 3.38±0.28b | | 4.78±0.07a |
|  |  | 100 | | 1.88±0.15c | | 1.04±0.14d | 2.76±0.05b | | 3.79±0.09a |
|  | Germination | | | 2.87±0.08c | | 2.47±0.06d | 3.06±0.05b | | 4.28±0.04a |
|  | Seeding | | | 3.81±0.09c | | 3.68±0.09d | 4.06±0.05b | | 4.44±0.04a |
|  | Sprouting | | | 4.27±0.16d | | 5.01±0.16c | 5.18±0.14b | | 6.15±0.26a |
|  | Flowering | | | 4.44±0.15d | | 5.19±0.16c | 5.48±0.16b | | 6.51±0.10a |
|  | Maturity | | | 3.19±0.06d | | 3.91±0.05c | 4.64±0.06b | | 5.66±0.17a |

**Table S3 Changes on available phosphorus content at 0‒100cm soil layers in different periods.**

| year | Fertile period | | Soil depth (cm) | CK | L | M | H |
| --- | --- | --- | --- | --- | --- | --- | --- |
| 2022 | Germination | | 20 | 15.78±1.29d | 18.38±1.23c | 22.60±0.49b | 35.43±1.01a |
|  |  |  | 40 | 12.37±0.97b | 6.04±0.49c | 13.83±2.12b | 20.00±1.23a |
|  |  |  | 60 | 8.48±0.49c | 6.85±1.1d | 10.1±0.28b | 12.21±1.01a |
|  |  |  | 80 | 5.07±0.49b | 8.8±0.56a | 9.94±1.69a | 10.42±0.97a |
|  |  |  | 100 | 7.34±2.68b | 8.80±1.23b | 12.05±0.74a | 14.00±0.74a |
|  | Seeding | | 20 | 68.55±2.50c | 62.54±1.29d | 85.11±2.50b | 96.47±1.97a |
|  |  |  | 40 | 57.18±0.84c | 37.86±0.74d | 74.55±2.20b | 80.89±2.30a |
|  |  |  | 60 | 23.57±2.53c | 29.26±0.74d | 32.02±1.84b | 43.38±1.23a |
|  |  |  | 80 | 13.33±2.55c | 21.79±0.74c | 20.98±2.98c | 29.26±0.74a |
|  |  |  | 100 | 13.67±3.94ab | 10.91±0.49b | 16.43±1.84a | 17.73±0.84a |
|  | Sprouting | | 20 | 46.47±1.76d | 49.39±4.25c | 63.35±4.43b | 71.79±2.96a |
|  |  |  | 40 | 25.36±1.01d | 31.04±1.01c | 47.6±3.97b | 60.43±3.09a |
|  |  |  | 60 | 10.59±1.01d | 19.25±0.74c | 23.19±1.95b | 33.32±3.04a |
|  |  |  | 80 | 7.99±0.97c | 11.72±1.57b | 13.18±1.57b | 20.98±3.69a |
|  |  |  | 100 | 6.36±0.74c | 7.66±0.28c | 8.31±1.23b | 12.05±0.74a |
|  | Flowering | | 20 | 50.04±3.46d | 64.06±1.97c | 76.02±2.5b | 90.95±1.71a |
|  |  |  | 40 | 23.57±1.46d | 41.92±2.45c | 50.53±3.52b | 60.27±1.41a |
|  |  |  | 60 | 11.72±1.49c | 23.03±1.41bc | 31.66±5.54b | 35.43±6.54a |
|  |  |  | 80 | 8.48±1.29d | 15.29±0.97c | 22.44±4.93b | 29.91±2.12a |
|  |  |  | 100 | 5.88±1.01c | 8.31±4.06c | 15.62±0.56b | 22.6±2.71a |
|  | Maturity | | 20 | 43.54±1.95c | 33.97±1.97d | 51.66±5.34b | 64.49±0.84a |
|  |  |  | 40 | 30.88±1.76c | 21.79±0.74d | 33.8±0.49b | 43.22±0.74a |
|  |  |  | 60 | 18.70±0.97b | 18.54±0.74b | 19.68±0.49b | 27.47±1.69a |
|  |  |  | 80 | 11.40±0.49b | 10.59±0.74b | 12.37±0.97b | 14.81±1.29a |
|  |  |  | 100 | 6.04±0.49b | 6.69±0.74b | 7.50±0.97b | 11.40±0.97a |
|  | Germination | | | 9.81±1.18c | 9.77±0.93c | 13.7±1.07b | 18.41±0.99a |
|  | Seeding | | | 35.26±2.47c | 32.47±0.8c | 45.82±2.27b | 53.55±1.42a |
|  | Sprouting | | | 19.35±1.1d | 24.61±1.57c | 30.33±3.03b | 39.71±2.71a |
|  | Flowering | | | 21.94±1.74d | 30.52±2.17c | 38.25±3.41b | 47.83±2.90a |
|  | Maturity | | | 20.11±1.13c | 18.31±0.99c | 25.00±1.65b | 32.28±1.11a |
| 2023 | Germination | 20 | | 29.91±1.01c | 31.2±1.70c | 33.79±0.74b | 38.48±1.48a |
|  |  | 40 | | 17.14±1.22c | 27.16±1.28b | 30.39±0.74ab | 28.45±1.22a |
|  |  | 60 | | 8.41±0.74c | 14.55±0.97b | 15.36±2.67b | 23.76±0.97a |
|  |  | 80 | | 5.17±0.74c | 10.02±0.74b | 10.35±1.01b | 14.23±0.74a |
|  |  | 100 | | 3.88±0.49c | 6.31±0.49b | 6.95±0.74b | 9.05±1.01a |
|  | Seeding | 20 | | 45.43±2.19c | 47.85±1.7b | 57.88±3.23b | 73.23±4.63a |
|  |  | 40 | | 36.37±1.94c | 38.96±2.19b | 49.95±3.03b | 55.45±2.92a |
|  |  | 60 | | 20.85±0.97d | 29.58±1.75c | 35.24±2.92b | 41.06±2.96a |
|  |  | 80 | | 13.42±0.74c | 13.9±0.74c | 21.02±2.02b | 25.54±0.74a |
|  |  | 100 | | 10.19±0.49b | 11.64±0.84b | 15.84±1.84a | 18.11±1.01a |
|  | Sprouting | 20 | | 21.99±0.74b | 21.99±0.74b | 23.60±1.48b | 28.61±0.97a |
|  |  | 40 | | 12.29±0.74c | 11.96±0.74c | 17.78±1.01b | 23.28±0.97a |
|  |  | 60 | | 8.73±0.49c | 9.22±0.49c | 10.99±1.01b | 15.84±1.22a |
|  |  | 80 | | 5.17±0.74c | 4.20±0.74c | 7.92±0.74b | 11.64±1.46a |
|  |  | 100 | | 3.72±0.28c | 2.59±0.56d | 6.31±0.49b | 8.41±0.74a |
|  | Flowering | 20 | | 40.74±0.84c | 32.33±0.28d | 57.55±5.52b | 64.02±2.22a |
|  |  | 40 | | 26.03±4.76b | 17.62±1.12c | 44.78±1.56a | 48.34±3.44a |
|  |  | 60 | | 15.68±1.01b | 12.13±1.28b | 25.38±2.84a | 29.26±3.23a |
|  |  | 80 | | 9.70±0.49b | 10.02±1.22b | 14.55±0.49a | 17.14±2.67a |
|  |  | 100 | | 5.82±0.49c | 6.47±0.28c | 8.08±0.56b | 10.19±0.49a |
|  | Maturity | 20 | | 31.52±1.46b | 25.87±1.01c | 32.82±1.01b | 40.58±2.29a |
|  |  | 40 | | 21.50±1.22b | 14.23±1.01c | 24.73±1.46a | 27.00±1.56a |
|  |  | 60 | | 7.60±0.28d | 10.19±0.84c | 12.77±0.56b | 17.30±1.48a |
|  |  | 80 | | 6.79±0.83b | 10.19±1.75ab | 9.22±1.94ab | 13.1±0.49a |
|  |  | 100 | | 4.69±0.56b | 4.04±0.28b | 4.90±2.48b | 9.05±1.22a |
|  | Germination | | | 12.9±0.84d | 17.85±1.04c | 19.37±1.38b | 22.79±1.09a |
|  | Seeding | | | 25.25±1.27d | 28.39±1.44c | 35.99±2.61b | 42.68±2.45a |
|  | Sprouting | | | 10.38±0.6c | 9.99±0.65c | 13.32±0.95b | 17.56±1.07a |
|  | Flowering | | | 19.59±1.52c | 15.71±0.84d | 30.07±2.19b | 33.79±2.41a |
|  | Maturity | | | 14.42±1.55c | 12.90±0.98d | 16.89±1.49b | 21.40±1.41a |

**Table S4 Changes on available potassium content at 0−100cm soil layers in different periods.**

| year | Fertile period | | Soil depth (cm) | CK | L | M | H |
| --- | --- | --- | --- | --- | --- | --- | --- |
| 2022 | Germination | | 20 | 130.92±2.03b | 126.17±0.35c | 147.20±1.47a | 151.51±1.21a |
|  |  |  | 40 | 117.15±5.58b | 112.97±2.84b | 134.70±2.29a | 136.02±2.31a |
|  |  |  | 60 | 101.1±2.12bc | 98.35±2.13c | 103.40±0.53b | 121.13±1.20a |
|  |  |  | 80 | 89.15±2.17bc | 82.59±1.74c | 91.95±2.80ab | 98.55±6.22a |
|  |  |  | 100 | 77.93±1.72bc | 73.98±1.68c | 81.59±5.91b | 89.96±3.64a |
|  | Seeding | | 20 | 171.41±6.2c | 167.85±2.09c | 188.86±5.91b | 198.19±4.86a |
|  |  |  | 40 | 154.34±2.88c | 151.85±5.40c | 174.48±1.51b | 188.25±0.57a |
|  |  |  | 60 | 139.62±4.59b | 123.25±5.21c | 146.65±2.08ab | 151.85±1.64a |
|  |  |  | 80 | 124.17±2.09ab | 119.08±2.27b | 125.49±2.11ab | 127.75±3.64a |
|  |  |  | 100 | 103.15±4.97b | 99.67±3.94b | 111.15±0.80a | 114.26±2.93a |
|  | Sprouting | | 20 | 116.67±4.52c | 110.30±4.90c | 138.31±4.30b | 148.71±2.41a |
|  |  |  | 40 | 103.26±1.36c | 98.79±1.74c | 115.55±3.65b | 135.00±5.70a |
|  |  |  | 60 | 99.31±3.49b | 90.17±0.93b | 110.89±7.37a | 114.6±2.43a |
|  |  |  | 80 | 92.30±3.83b | 86.72±0.63b | 106.64±3.87a | 109.85±1.21a |
|  |  |  | 100 | 80.11±2.81b | 75.39±0.52c | 90.15±2.16ab | 96.85±2.28a |
|  | Flowering | | 20 | 202.35±4.39b | 197.41±3.17b | 207.61±4.13b | 233.87±5.07a |
|  |  |  | 40 | 170.03±2.22c | 160.4±2.17d | 186.57±4.00b | 201.05±4.90a |
|  |  |  | 60 | 152.19±6.34c | 150.65±4.84c | 167.31±2.12b | 175.95±0.54a |
|  |  |  | 80 | 132.32±6.9bc | 128.89±9.73c | 141.55±1.8ab | 149.28±4.44a |
|  |  |  | 100 | 119.95±1.37ab | 106.97±7.02b | 115.23±4.59b | 127.63±1.35a |
|  | Maturity | | 20 | 154.81±3.39c | 148.95±5.33c | 168.57±4.40b | 180.41±5.09a |
|  |  |  | 40 | 136.82±3.24c | 126.86±2.16c | 147.86±3.21b | 162.68±1.86a |
|  |  |  | 60 | 118.39±2.52c | 116.54±4.16c | 140.53±3.77b | 155.15±2.62a |
|  |  |  | 80 | 108.33±1.38b | 105.45±4.24b | 134.55±4.55a | 141.34±0.24a |
|  |  |  | 100 | 94.57±5.03bc | 88.17±3.08c | 106.37±4.23ab | 117.88±1.09a |
|  | Germination | | | 117.26±2.73c | 120.74±1.52d | 133.97±1.42b | 126.95±2.95a |
|  | Seeding | | | 140.32±2.57c | 149.33±2.48d | 154.04±2.28b | 130.72±3.06a |
|  | Sprouting | | | 91.07±1.74c | 112.31±4.27d | 121.00±2.81b | 98.33±3.20a |
|  | Flowering | | | 146.02±4.3c | 167.59±4.24d | 179.89±3.95b | 154.99±4.85a |
|  | Maturity | | | 117.19±3.79c | 139.58±4.03d | 151.49±2.18b | 116.45±2.92 |
| 2023 | Germination | 20 | | 119.32±4.97b | 105.05±1.26c | 132.55±1.27a | 142.33±4.39a |
|  |  | 40 | | 111.25±2.14b | 94.39±0.57c | 113.18±2.92b | 129.38±0.29a |
|  |  | 60 | | 94.30±2.14b | 86.76±2.95c | 106.01±2.34a | 108.98±3.49a |
|  |  | 80 | | 86.31±2.34c | 80.42±2.61d | 92.16±1.66b | 100.16±0.29a |
|  |  | 100 | | 79.48±1.07b | 70.00±3.01c | 81.62±3.70b | 92.23±7.80a |
|  | Seeding | 20 | | 172.27±3.57c | 158.21±4.12d | 192.25±5.85b | 208.19±4.98a |
|  |  | 40 | | 149.98±4.97c | 148.05±4.73c | 169.23±2.89b | 183.02±0.35a |
|  |  | 60 | | 133.58±2.77c | 131.33±0.44c | 147.69±3.42b | 155.4±3.56a |
|  |  | 80 | | 118.24±1.89b | 108.96±1.11c | 127.15±1.36a | 130.60±1.30a |
|  |  | 100 | | 106.15±3.84b | 102.71±0.84b | 115.3±3.47a | 119.11±2.26a |
|  | Sprouting | 20 | | 131.97±3.30c | 123.8±4.41d | 153.34±3.58b | 161.79±5.68a |
|  |  | 40 | | 124.25±4.96c | 117.13±3.42d | 144.56±0.35b | 150.53±4.37a |
|  |  | 60 | | 103.12±4.14c | 98.8±0.56d | 124.53±1.55b | 135.92±4.97a |
|  |  | 80 | | 95.68±1.24c | 85.29±3.39d | 109.14±3.91b | 114.88±4.52a |
|  |  | 100 | | 83.96±2.59c | 75.00±3.76d | 96.87±2.58b | 103.44±1.94a |
|  | Flowering | 20 | | 145.39±4.42c | 137.76±2.14d | 175.53±3.23b | 186.6±6.47a |
|  |  | 40 | | 135.56±3.65c | 129.31±5.41d | 149.98±3.35b | 159.63±1.04a |
|  |  | 60 | | 122.37±0.35c | 113.18±1.8d | 130.18±3.66b | 144.7±1.77a |
|  |  | 80 | | 110.89±1.34b | 102.52±1.54b | 123.61±3.90a | 127.56±6.45a |
|  |  | 100 | | 94.76±2.83b | 92.42±2.22b | 107.72±2.24a | 111.25±2.45a |
|  | Maturity | 20 | | 125.45±0.55c | 121.64±2.02c | 147.92±3.15b | 161.56±1.82a |
|  |  | 40 | | 107.85±1.11c | 110.61±4.98c | 131.84±3.47b | 142.54±3.77a |
|  |  | 60 | | 101.15±1.07c | 97.56±2.84c | 114.93±5.96b | 124.35±2.53a |
|  |  | 80 | | 86.77±2.01c | 83.14±1.90c | 106.47±0.90b | 113.32±1.04a |
|  |  | 100 | | 81.53±2.56c | 77.94±2.24c | 96.41±1.51b | 105.28±1.52a |
|  | Germination | | | 98.13±2.53c | 87.33±2.08c | 105.11±2.38b | 114.62±3.65a |
|  | Seeding | | | 136.04±3.81c | 129.85±3.85d | 150.32±1.60b | 159.26±4.09a |
|  | Sprouting | | | 107.8±1.25c | 100.01±4.91d | 125.69±4.99b | 133.31±3.89a |
|  | Flowering | | | 121.79±5.12c | 115.04±1.62d | 137.4±3.28b | 145.95±5.84a |
|  | Maturity | | | 100.55±1.46c | 98.18±1.99c | 119.51±2.40b | 129.41±4.54a |

**Table S5 Changes on soil nitrate nitrogen content at 0−100cm soil layers in different periods.**

| year | Fertile period | | Soil depth (cm) | CK | L | M | H |
| --- | --- | --- | --- | --- | --- | --- | --- |
| 2022 | Germination | | 20 | 5.95±0.56c | 3.71±0.46d | 8.82±0.25b | 10.23±0.22a |
|  |  |  | 40 | 3.53±0.05c | 2.91±0.04d | 4.90±0.09b | 5.95±0.07a |
|  |  |  | 60 | 2.04±0.06c | 1.96±0.28c | 2.58±0.06b | 5.13±0.11a |
|  |  |  | 80 | 1.61±0.170b | 1.76±0.06b | 1.76±0.16b | 3.83±0.71a |
|  |  |  | 100 | 1.06±0.03c | 1.00±0.02c | 1.30±0.02b | 1.95±0.07a |
|  | Seeding | | 20 | 10.12±0.09c | 9.95±0.97c | 14.45±1.01b | 18.9±0.90a |
|  |  |  | 40 | 7.25±0.96c | 6.69±0.94c | 9.67±0.10b | 14.98±0.64a |
|  |  |  | 60 | 5.25±0.96c | 4.43±0.65d | 6.88±0.54b | 9.36±0.56a |
|  |  |  | 80 | 3.28±0.04c | 2.81±0.04d | 4.43±0.1b | 7.48±0.86a |
|  |  |  | 100 | 3.05±0.06d | 2.52±0.64c | 3.98±0.39b | 4.48±0.76a |
|  | Sprouting | | 20 | 5.32±0.58c | 5.06±0.56c | 8.68±0.5b | 11.22±0.73a |
|  |  |  | 40 | 4.16±0.46c | 3.46±0.39d | 5.61±0.45b | 6.17±0.15a |
|  |  |  | 60 | 2.97±0.46c | 2.93±0.05c | 4.36±0.36b | 4.73±0.17a |
|  |  |  | 80 | 2.45±0.37c | 2.05±0.34d | 2.89±0.22b | 3.18±0.04a |
|  |  |  | 100 | 1.23±0.14b | 1.04±0.04b | 2.22±0.25a | 2.56±0.10a |
|  | Flowering | | 20 | 6.52±0.94c | 5.98±0.59c | 11.13±0.71b | 16.94±1.06a |
|  |  |  | 40 | 4.76±0.66c | 3.67±0.47d | 7.87±0.85b | 9.17±0.85a |
|  |  |  | 60 | 3.19±0.11c | 2.78±0.48c | 4.79±0.46b | 7.73±0.67a |
|  |  |  | 80 | 2.43±0.33c | 2.08±0.35c | 3.91±0.43b | 5.56±0.10a |
|  |  |  | 100 | 1.19±0.31b | 1.04±0.23b | 2.42±0.19a | 3.02±0.49a |
|  | Maturity | | 20 | 6.41±0.86c | 5.73±0.87d | 8.86±0.14b | 11.34±0.67a |
|  |  |  | 40 | 4.85±0.57c | 4.04±0.54d | 6.44±0.06b | 8.35±0.37a |
|  |  |  | 60 | 3.42±0.4c | 2.81±0.33d | 5.08±0.12b | 6.67±0.67a |
|  |  |  | 80 | 2.4±0.36b | 2.35±0.14b | 3.16±0.58a | 3.38±0.42a |
|  |  |  | 100 | 1.57±0.23c | 1.36±0.08d | 1.94±0.07b | 2.38±0.02a |
|  | Germination | | | 2.84±0.41d | 2.27±0.3c | 3.87±0.64b | 6.02±0.20a |
|  | Seeding | | | 4.59±0.33c | 3.78±0.21d | 7.48±0.94b | 11.04±0.35a |
|  | Sprouting | | | 3.23±0.52c | 2.91±0.42d | 4.55±0.43b | 5.57±0.65a |
|  | Flowering | | | 3.62±0.32d | 3.11±0.02c | 6.03±0.51b | 8.49±0.74a |
|  | Maturity | | | 3.73±0.73d | 3.26±0.53c | 5.09±0.03b | 6.43±0.03a |
| 2023 | Germination | 20 | | 7.42±0.48bc | 7.18±0.18bc | 8.19±0.29b | 10.11±0.48a |
|  |  | 40 | | 6.00±0.17b | 5.88±0.15b | 6.01±0.42b | 6.82±0.32a |
|  |  | 60 | | 4.10±0.17b | 3.90±0.23b | 3.72±0.51b | 4.64±0.13a |
|  |  | 80 | | 2.66±0.27b | 2.41±0.22b | 3.09±0.14b | 3.64±0.34a |
|  |  | 100 | | 1.69±0.25c | 1.61±0.39c | 2.26±0.38b | 2.82±0.20a |
|  | Seeding | 20 | | 13.55±0.55b | 10.3±0.63c | 16.8±0.33a | 17.18±0.42a |
|  |  | 40 | | 9.18±0.47b | 9.52±0.09b | 14.72±0.05a | 15.01±1.19a |
|  |  | 60 | | 6.99±0.53b | 6.46±0.20b | 6.50±0.130b | 8.68±0.13a |
|  |  | 80 | | 5.17±0.24d | 5.93±0.13a | 5.31±0.23c | 5.63±0.32b |
|  |  | 100 | | 3.97±0.20b | 3.67±0.12d | 4.69±0.44a | 3.54±0.32c |
|  | Sprouting | 20 | | 9.15±0.09b | 9.06±0.41b | 11.60±0.52a | 11.33±0.01a |
|  |  | 40 | | 6.12±0.42b | 6.07±0.49b | 10.73±0.46a | 10.86±0.02a |
|  |  | 60 | | 5.11±0.28b | 4.97±0.29b | 8.34±0.31a | 8.58±0.18a |
|  |  | 80 | | 3.40±0.31b | 3.08±0.58b | 5.89±0.31a | 6.30±0.47a |
|  |  | 100 | | 2.04±0.35c | 1.78±0.43c | 3.60±0.02b | 4.86±0.12a |
|  | Flowering | 20 | | 9.04±0.35b | 8.89±0.50b | 10.32±0.58a | 12.07±0.63a |
|  |  | 40 | | 6.63±0.70b | 6.43±0.12b | 7.92±0.31a | 8.77±0.34a |
|  |  | 60 | | 5.59±0.20b | 5.46±0.36b | 5.62±0.02a | 6.24±0.03a |
|  |  | 80 | | 4.48±0.29b | 4.34±0.03b | 4.67±0.02a | 4.89±0.23a |
|  |  | 100 | | 3.08±0.20c | 2.88±0.29c | 3.54±0.39a | 3.80±0.51a |
|  | Maturity | 20 | | 9.91±0.29c | 9.11±0.40d | 10.9±0.71b | 11.9±0.42a |
|  |  | 40 | | 7.10±0.35c | 7.01±0.36c | 8.66±0.58b | 9.09±0.54a |
|  |  | 60 | | 5.49±0.36c | 4.90±0.42d | 6.06±0.66b | 6.33±0.88a |
|  |  | 80 | | 3.73±0.11b | 3.59±0.31b | 4.67±0.56a | 5.34±0.17a |
|  |  | 100 | | 2.28±0.60c | 2.04±0.32d | 3.22±0.64b | 3.86±0.60a |
|  | Germination | | | 4.38±0.26c | 4.2±0.23d | 4.66±0.11b | 5.60±0.11a |
|  | Seeding | | | 7.77±0.08b | 7.18±0.11b | 9.60±0.24a | 10.01±0.48a |
|  | Sprouting | | | 5.16±0.29b | 4.99±0.44b | 8.03±0.07a | 8.39±0.16a |
|  | Flowering | | | 5.77±0.35c | 5.6±0.26d | 6.41±0.26b | 7.15±0.35a |
|  | Maturity | | | 5.70±0.34c | 5.33±0.36d | 6.70±0.63b | 7.30±0.52a |
